# Beyond the scars: a qualitative study on the experiences of mastectomy among young women with breast cancer in a country with crisis

**DOI:** 10.1186/s12905-023-02734-0

**Published:** 2023-11-13

**Authors:** Shaza Hasan, Keng Sheng Chew, Rekaya Vincent Balang, Shirly Siew Ling Wong

**Affiliations:** 1 Faculty of Medicine and Health Sciences, Universiti Malaysia Sarawak, 94300 Kota Samarahan, Sarawak Malaysia; 2 Faculty of Economics and Business, Universiti Malaysia Sarawak, 94300 Kota Samarahan, Sarawak Malaysia

**Keywords:** Breast cancer, Mastectomy, Young women, Syria, Crisis

## Abstract

**Background:**

As breast cancer incidence rises among younger women, there is a knowledge gap regarding the emotional, physical, and social effects of mastectomy, specifically in a crisis-affected country such as Syria. This study aimed to explore these effects on young women with breast cancer in Syria, taking into consideration the cultural significance of a woman’s breast as part of her feminine identity.

**Methods:**

A qualitative design, using semi-structured in-depth interviews with 10 young women with breast cancer who underwent mastectomy, was conducted between June to December 2022.

**Results:**

Thematic analysis was used to analyze the data, and five main themes were identified: (1) psychological and emotional well-being (altered self-esteem and femininity, impact on sexual life and relationships, psychological distress associated with mastectomy, mirror trauma and the need for psychological care); (2) body image and breast reconstruction (the dilemma over reconstruction decision, body image and clothing and lack of access to prosthetic information/services); (3) social and interpersonal factors (lack of marriage choices and society’s view and stigma); (4) coping mechanisms with mastectomy effects (family support; faith in god almighty; comparing their situation to others and use of prosthetics) and (5) physical health and functioning (physical effects on mobility and function).

**Conclusion:**

Mastectomy has significant physical, emotional, and social consequences on young women with breast cancer, particularly in crisis-affected Syria where access to breast reconstruction is limited. It is crucial for healthcare professionals to understand these impacts, to raise awareness, encourage early detection, and promote less aggressive treatments to improve women’s quality of life.

## Background

Breast cancer is the most common cancer and a leading cause of death among women worldwide [1]. Historically, breast cancer was known as a disease of postmenopausal women [2]. However, its incidence has significantly increased among younger women in recent decades. In Arabic countries, breast cancer accounts for 14—42% of all female cancer cases, with approximately half of these cases occurring in women under 50, in contrast to 25% in developed countries [3]. In Syria, the most recent data from the Syrian National Cancer Registry was published in 2009, but no report has been published since [4, 5], due to the constraints on the registry’s role and function after the start of the country’s crisis. However, a report from the World Health Organization in 2020 showed that breast cancer was still ranked top among types of cancers affecting women in Syria with 4388 new reported cases in 2020 [6].

The woman’s breast is often considered a symbol of her identity in Syria and in other countries. It represents her femininity, sexuality, beauty, and motherhood [7, 8]. Therefore, removing one or both breasts by mastectomy can adversely affect women emotionally, physically, and socially, especially among younger women as younger women are still establishing their education, careers, and families [9–11]. These adverse effects include distorted body image perception, loss of self-confidence, loss of self-esteem and sense of femininity, and changes in relationships and social life. These negative effects can be exacerbated by the negative societal view toward cancer patients, as cancer is still recognized as a stigma and a death sentence in many cultures including the Arabic culture [12, 13]. The relevant literature emphasizes that young women with early-stage breast cancer tend to choose mastectomy over breast-conserving surgery due to fear of recurrence [14, 15], despite the more favorable outcomes of conserving surgery compared to mastectomy as demonstrated in a meta-analysis [16].

The cancer care in Syria has significantly deteriorated since the start of the crisis in 2011. Indeed, the Syrian crisis has resulted in many challenges affecting the quality of care and life of cancer patients. These challenges included the unavailability of a multidisciplinary team approach to cancer care, the high cost of different types of treatment, the insufficiency of health staff training for diagnosis, treatment, palliative care, and psychological care of cancer patients, as well as the lack of the educational programs designed for patients and oncology health professionals [4]. All these factors have led to suboptimal care for Syrian women with breast cancer.

Unfortunately, the current literature lacks information on the effects of mastectomy on young women with breast cancer in a crisis-affected country like Syria. Furthermore, breast cancer is a complex health issue, with repercussions that may extend beyond the immediate patient, but affecting her familial and spousal relationships as well [17]. For example, within a marital context, breast cancer has been shown to precipitate sexual dysfunctions, endured by both the women themselves and their spouses. These predicaments are rendered particularly salient and nuanced within the male-dominated socio-cultural Middle Eastern Muslim context, where female passivity in sexual relationships is normative, and overt female expressions of sexual desire are considered culturally inappropriate [17, 18]. Consequently, many of these challenges encountered by Syrian women with breast cancer may remain hidden beneath the veneer of cultural shroud. Therefore, this study was conducted with the aim to explore the effects of mastectomy on young women with breast cancer in Syria. By understanding these effects on young women, appropriate post-mastectomy educational and intervention programs to support and prepare these patients psychologically and socially can be implemented.

## Methods

### Study design and participants

This study, which is part of a larger qualitative study undertaken to explore the psychosocial needs of young women with breast cancer in Syria, was conducted using a phenomenological approach to understand the mastectomy phenomenon through detailed explorations of the experiences of these participants.

Prior to commencing the study, ethical approval was obtained from the institutional medical ethics committee of University Malaysia Sarawak (No: FME/22/42), and approval was also granted by the Department of Oncology of Tishreen University Hospital. Informed consent was also obtained from the participants that they agree to participate in the study.

### Participants

Purposive sampling method was used to recruit the participants from Tishreen University Hospital in Latakia, Syria, which is the second most comprehensive oncological center in Syria and receives oncological patients from many cities around Latakia. Purposive sampling means that researchers intentionally recruit participants who have experienced the central phenomenon, or the key concept being explored in the study [19]. Participants were included if they were aged between 18–50 years (defined as “young” in this study), had histologically proven new breast cancer with no evidence of recurrence, underwent mastectomy and were proficient in Arabic language. Those with evidence of recurrences and metastases were excluded. The recruitment process was iteratively continued until data saturation was believed to have been achieved where no new findings and insights could be generated.

### Data collection

After recruitment, the participants were contacted by the first author to arrange for the time and place of the interview sessions. The in-depth, semi-structured interviews were conducted according to the participants’ time in a private room at home to allow the participants to freely answer the questions. The interviews were conducted using an interview guide consisting of these questions: (1) Can you please describe your experience with mastectomy? (2) How did you cope with the mastectomy? (3) How do you see the view of Syrian society towards women with breast cancer? (4) How did a mastectomy affect your sexual relationship with your husband? (5) Do you plan to go for breast reconstruction? These questions were developed by all the authors based on their experiences and the existing literature [7, 17–19]. The interview guide was first developed in English and then translated into Arabic and pilot-tested on one young woman with breast cancer to check the feasibility, validity, and ease of comprehension. All the interviews were conducted in Arabic (between June and December 2022) by the first author, who is an Arabic Muslim and an Arabic native speaker. The interviews lasted between 30 and 60 min, and permission to record the interview was obtained from the participants before the interview started. Demographic and medical questions such as age, marital status, having children, education status, religion, monthly income, employment, and time since diagnosis (months) were collected during interview sessions.

### Data analysis

All the interviews were audio recorded, immediately transcribed verbatim by the first author, and translated into English by the first author to ensure accessibility and comprehension for all authors, as they are not Arabic speakers except for the first author. Thematic analysis was used to analyze the data, using the six-phases approach outlined by Braun and Clarke [20]. Thematic analysis is a method for identifying, analyzing, and reporting patterns (themes) within data. These six phases included: data familiarization, generating initial codes, searching for themes, reviewing themes, defining and naming themes, and producing the report. NVivo 10 was used for the data analysis. The coding process was done by the first author and the second author (who is a Malaysian Chinese Christian) separately. Each author established the initial themes independently, and then these initial themes were discussed, reviewed, refined, and labelled by both authors to reach a consensus for the final themes. Regarding trustworthiness and credibility, this was established through member checking. Member checking was done by asking the participants to verify the transcribed data. A third author, who was an expert in qualitative research, was asked to review and assess the transcripts, data analyses, and findings.

## Results

As shown in Table 1, the participants ranged in age from 28 to 48 years. Seven participants were married, 2 were widowed, and 1 was single. All of the participants had children, except the single participant. Four participants were at the university level of the education, 4 were at the institute level (2 years after secondary certificate), 1 at the secondary, and 1 at the primary level. Four participants were housewives and 6 were working. Time since diagnosis ranged from 10 to 36 months. The monthly income was less than $50 for 4 of them, less than $75 for 5 of them, and less than $100 for 1 of them. Five main themes with subthemes were identified from the analysis; these themes are summarized in Table 2.

**Table 1:** Demographic characteristics of the participants (*n* = 10)

Characteristics	N
**Age (years)** (*M* = 41.20, SD = 6.125**)**	
Less than 30	1
31- 40	4
41- 48	5
**Marital status**	
Married	7
Single	1
Widow	2
**Having children**	
Yes	9
No	1
**Education**	
Primary school	1
Secondary school	1
Institution (2 years after secondary school)	4
University	4
**Work**	
Housewives	4
Working	6
**Time since diagnosis**	
3- 12 months	6
13–24 months	2
25–36 months	2
**Monthly income**	
Less than $50	4
$51- $75	5
$76- $100	1

**Table 2:** Themes and subthemes identified from the analysis

Themes	Subthemes
(1) Psychological and Emotional Well-Being	• Altered self-esteem and femininity • Impact on sexual life and relationships • Psychological distress associated with mastectomy • Mirror trauma • The need for Psychological Care
(2) Body Image and Breast Reconstruction	• The dilemma over reconstruction decision • Body image and clothing • Lack of access to prosthetic information/services:
(3) Social and Interpersonal Factors	• Lack of marriage choices • Society’s view and stigma
(4) Coping Mechanisms with Mastectomy Effects	• Family support • Faith in God Almighty • Comparing their situation to others • Use of prosthetics
(5) Physical Health and Functioning	• Physical effects on mobility and function

### Theme 1: psychological and emotional well-being

#### Altered self-esteem and femininity

Six out of 10 participants expressed the feelings of losing the breast as equivalent to losing part of their femininity after the mastectomy, which in turn, affected their self-esteem and body image. They mentioned the importance of maintaining the appearance of their breasts through the use of fillings and bras. For example, the participants said:

*“A woman loses her femininity when she does such an operation (mastectomy), she loses her beauty from her perspective, and when she loses her beauty, she doubts her beauty in front of people”.—H*

*“Look, the lady really misses a feminist part from her body, I think she feels there is something missing. She loses self- confidence, specifically if she is married. Life got more exhausting; you have to take care of the issue of the breast by putting pads. It becomes like a burden and you have to attend to it”.—RA*

#### Impact on sexual life and relationships

The participants expressed mixed feelings regarding the effects of mastectomy on their sexual relationships. Some avoided sexual contact because they felt shy, incomplete, and unattractive with only one breast, while others felt that their partners were supportive and understanding enough to help them cope with these challenges. When asked if mastectomy affected their relationships with their husbands, some participants answered:

*“For me, yes, but for him, no. I no longer want to have sexual intimacy, and I try to avoid it. I have always tried to hide the wound from him, but he says, ‘Let me see. I want to see how the wound has healed.’ He wants to tell me that he has no problem with it. He told me that as soon as I came out of surgery, while I was still under anesthesia. The first thing I asked was if he had seen me without a breast. Even when I was unconscious, I asked about this point. This shows how much this subject affects me, and I have no idea about that.” – RA.*

*“I was distant from my husband sexually, especially in the beginning, and praise be to God, he accepted the situation and encouraged and supported me.” – Ri*

*“No, not at all, thank God. My husband understands my situation, and he has never been affected by all these things. Especially when he sees me getting tired, he never forces me or tries to get close to me. Our relationship is usual, with foreplay, and our life is very normal.” – L.*

#### Psychological distress associated with mastectomy

Most of the participants expressed that the mastectomy experience was very difficult and distressing. Some of them mentioned that it was very difficult to overcome this experience and its consequences, and others mentioned that they were unable to overcome it yet. In turn, this psychological impact affected their daily lives and interactions with others. For example, some participants said:

*“But the breast loss is more distressing than hair loss, it is very painful and till now it is still painful”- RA*

*“It was very annoying, I can’t tell you what it can do with the female psyche, thank God”- G*

#### Mirror trauma

Four out of 10 participants mentioned the difficulty of viewing the mirror after the mastectomy. They mentioned that they avoided looking at themselves in front of the mirror or during the showers in order not to see such a valuable feminist organ missing from their bodies and feel upset. For example, the participants said:

*“It is very ugly. Till now I sometimes find it hard to accept this and I avoid looking at myself. Even in the bathroom.”- W*

*“When you look at yourself in the mirror and see your feminist part is missing, you feel upset. I was crying and saying, Oh my Lord, why me”? L*

*“When I was passing near the mirror, I was not looking at it at all.”- W*

#### The need for psychological care

The majority of the participants expressed that they had not received adequate psychological care at the hospital, highlighting the need to have an in-house psychologist in the treatment plan of cancer patients specifically in addressing these distressing conditions where they live. They mentioned that the psychologist could play a positive role in supporting the patients psychologically and socially. For example, one of the participants said when she was asked, what are the needs you feel should be available at the hospital to feel that you had received adequate psychological care.

*“There should be a psychologist to support the patients both psychologically and socially”- L*

Another participant said:

*“The psychologist is very helpful. For example, you can tell him things that you might not feel comfortable sharing with your doctor, husband, or anyone else in your family. He can be the keeper of your secrets. I am eagerly waiting to go to the hospital to meet the psychologist and tell him what I need. It is comforting to talk to him about the situations that have happened to you.” – A.*

Another participant also highlighted the need for psychologists by saying:

*“To have a psychologist is a very important thing, but unfortunately, this is not followed here due to a lack of psychologists, which could adversely affect us. The patient needs a companion, and the companion should never be from the relatives. He should be a psychologist, in my opinion.” H.*

### Theme 2: body image and breast reconstruction

#### The dilemma over reconstruction decision

Six of the participants expressed the desire to undergo breast reconstruction, noting that it could improve their psychological well-being and enable them to wear clothes they feel comfortable in. However, they mentioned that they are unable to restoring their pre-mastectomy body shape due to the high cost of reconstruction compared to their current financial status resulting from the ongoing crisis that has affected all the life aspects. For example, when asked on what they thought on reconstruction, some participants said:

*“Psychologically and physically, if I do the reconstruction, I may no longer feel ashamed, there are many t-shirts I can no longer wear them now because they are in open tops, I can no longer wear them if I don’t wear something higher under them, because it would look very bad. Even at the parties, I can’t wear what I want…. But the operation is very expensive, about $1000, it is impossible. It is a very big financial burden”- W*

*“I want $1000 to reconstruct this breast and $1000 for the second breast because I want to do evacuation and filing, means I need $2000, and this is not easy at all”- A*

On the other hand, few participants expressed the decision that they have no desire to do reconstruction because they are scared from its side effects including the misconception that reconstruction would cause the cancer to recur*.* For example, the following participant said:

*“No, I don’t think of doing plastic surgery because I am afraid of the side effects later on. I am afraid that it will be a reason for the disease to return again. Scientifically, I do not know if it could be a reason, but I would say that I do not have to do it, maybe if I am younger, I can think about it, but at this age I want to complete my life with my children, this is my only concern”- R.*

#### Body image and clothing

Some participants also shared about their struggles with adjusting to their new body image and finding appropriate clothing post-mastectomy, as described by these two participants:

*“As for the clothes, I was feeling uncomfortable, I underwent the surgery in the winter and this helped me a lot really because I was wearing loose jackets that were hiding. I was unable to wear the bra, especially when the surgery had just been done. Once, I was gonging for a walk, I tried to wear a bra but I could not, then I put on a loose jacket. I was wondering, how would I wear the summer clothes? I think women suffer more in summer than winter. Now the wound is healing and I wear a normal bra and I put padding in it”- R.*

*“I am one of those people who sometimes feel upset, because I like to wear a nice shirt, but because I am a patient with breast cancer, I find difficulty to fit the bra and the fillings, so I cancel the idea and say it would not be sweet on me to wear that shirt”- D.*

#### Lack of access to prosthetic information/services

Half of the participants detailed the challenges they encountered in acquiring information and resources related to breast prosthetics, emphasizing the necessity for improved access to these services within their region. For instance, when asked if they were informed about the available services at the hospital to assist them in enhancing their appearance, one participant stated:

*“No, never. I have not heard about that at the hospital; we really need a lot of information. Recently, I have heard that there is a center in Latakia that provides free bras for women who have undergone mastectomy. I only heard about this one month ago, and I had no prior knowledge of this center, and I haven’t visited it yet”- R.*

Another participant recounted her experience in searching for a silicone breast as follows:

*“Do you know that I searched throughout Damascus to find a silicone breast, and when I finally found it, it was in a larger size and without a carrier? In Latakia, I could not find it anywhere. There should be a center in each governorate, or at least in three governorates in Syria, or in two – on the coast and in Damascus. There should be two comprehensive centers to provide services for both women and men in such circumstances”- H.*

### Theme 3: social and interpersonal factors

#### Lack of marriage choices

Some unmarried participants lamented their concerns about the gloomy marital prospects post-mastectomy, worrying about how their future partners would perceive their physical appearance. For example,

*“It is possible that I or any other woman thinking of getting marriage, but now she may not think of that anymore. Why? Because who wants to marry a sick woman, who has undergone a mastectomy and she will be in a long- term treatment, and she has become deformed in her body? I will be ashamed of exposing myself to a man. He may not accept me, and he may be angry at me at once and stigmatize me for my illness, because in our culture, this is bad for men, friends, and family”- H.*

Another participant said:

*“I told my fiancé if they are going to do mastectomy, I will break up with you, I couldn’t let you see me distorted, if I cannot accept my appearance like this, how will he accept me?” W.*

#### Society’s view and stigma

All the participants talked about the negative society view and stigmatization of patients with breast cancer. They reported that society often focused on their chest area and showed curiosity about their condition. They also mentioned experiencing pity and unsolicited comments from others, which further contributed to their distress.

*“I sometimes get denounced by outsiders that I did the mastectomy. For example, I hear comments like, it is good that your husband is still willing to touch you, or it is good that he is not disgusted by your appearance”- A.*

*“The only thing that made me sad was that I felt ashamed whenever I went outside of the house because anyone who knows what I have gone through would immediately focus on my chest and that makes me feel sad that my breast was removed and that I am a cancer patient. That was what I had to learn to cope with”- D.*

### Theme 4: coping mechanisms with mastectomy effects

Despite the emotional distress from losing the breasts by mastectomy, some of the participants tried to accept and cope with their new situation by using different coping strategies, while others were unable to do so.

#### Family support

Some participants highlighted the vital roles their partners and family members played in providing emotional support during their breast cancer journey. For example, the participant said:

*“At the first shower, the whole family was waiting for me at the door…this helped me really. There was my husband, my children and my mother, and I felt they are waiting for me outside, so I know I have to be strong and this helped me a lot, this helped me a lot really. My children keep asking, do you need anything mom? They just want to know my situation inside”-R.*

#### Faith in God Almighty

All the participants expressed that engaging in the spiritual practices such as offering the prayer and reading the Quran, and comparing themselves with those who were in worse situation helped them to accept their situation easier. They repeatedly expressed their strong faith in “God Almighty” taking the Quran and the religious as their shelter when they feel weak and stress. For example, a participant said:

*“The first shower was very difficult, but I didn’t care, I tried to help myself, I said it is something that had happened, I have to accept, and I wanted to live for my children. There are many people are suffering more and worse than this, there are people waiting to die, I mean, I kept helping myself with prayers and reading Al- Quran. This helped me a lot”- R.*

#### Comparing their situation to others

Some participants mentioned that comparing their situation to others who may be worse off helped them to accept as said by this participant:

*“Now I am suffering the discomfort in my hand and I can’t work that much because of it. But I say, praise be to God, it is the left hand, because I see someone else in suffering at her right hand, so I say, it should be easier for me to handle, praise be to God”-RO.*

#### Use of prosthetics

In addition to that, the participants also tried to cope by using the pads and fillings to hide the shape of the missing breast. For example, when asked how you tried to cope with mastectomy, one of the participants said:

*“I don’t know, it was very difficult, but I encouraged myself that it could be compensated by implants, at least for a while, and hopefully it will pass. I made a hand making filling, I first got a silicone breast, and even this thing is not available”- H.*

### Theme 5: physical health and functioning

#### Physical effects on mobility and function

Seven of the participants reported physical limitations due to the surgery, such as reduced hand mobility and the need for assistance in daily activities**.** They mentioned that these effects were not only during the acute phase post-mastectomy, but they became long- lasting effects limiting of their activities as described by the participants below:

*“I was more active, there are many things I can’t do because of my hand, is it right or not? This is what is bothering me that I can’t work like before”- RI*

*“I need someone to help me at home. It is obligatory, I cannot do everything by myself. I find some difficult to do many things, I can do it but I feel uncomfortable”- RO*

Some participants also reported that they felt guilty over their shortcomings towards their children because, they were unable to cook for them after the mastectomy, and some of them depended on their children a lot to do the household chores after the surgery. For example, some participants said:

*“They (the children) were very upset, sad and got busy at home a lot, because before that, I did not have to rely on them a lot, may be like 20 or 30% of the chores only I asked them to do. But now I rely on them a lot in the household chores, especially because of my hand. The children really helped me a lot”- R*

*“It was Ramadan. I couldn’t cook the foods they like while they were fasting. This affected me a lot psychologically, but then, thank God, now I am coming back a little by little, not fully functioning like before, but at least, a dish that they like, like sweet food something like that”- Ri*

## Discussion

**Fig. 1:**
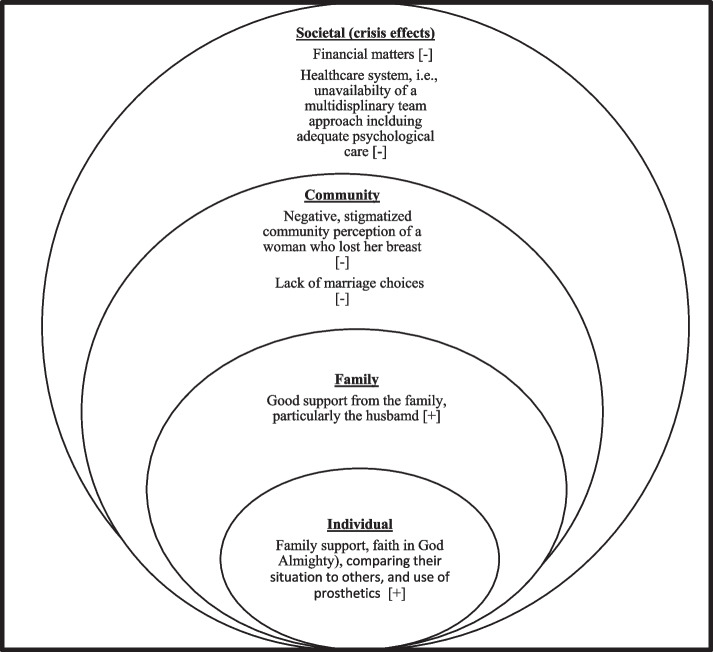
The Social- Ecological Model Depicting Various Facilitative [ +] and Obstructive Factors [-] Affecting the Capacity to Cope with the Effects of Mastectomy

The findings from this study suggest that mastectomy yields far-reaching consequences that can significantly impact various aspects of a woman’s life in a country experiencing a crisis; encompassing the physical, emotional, and social dimensions. For example, a substantial proportion of participants reported experiencing a diminished sense of femininity, an altered perception of their body image, and a decreased self-esteem as a consequence of undergoing mastectomy, which ultimately result in feeling of shame and perceived disfigurement. Moreover, the participants also shared a range of facilitative and obstructive factors that respectively promote and impede their capacity to manage the repercussions of mastectomy. We posit that these factors can be appropriately fit into the socio-ecological model of health [21]. First proposed by Bronfenbrenner (1977), socio-ecological model is a multi-faceted framework depicting the complex interplay between personal, family, community, and societal determinants of health and illness. In this regard, the personal and family factors in our study are predominantly facilitative in nature whereas, the community and societal factors are predominantly obstructive in nature (see Fig. 1).

In Syrian and other similar cultures, a woman’s breast represents her femininity [7, 8], hence, mastectomy can negatively affect a woman’s self-confidence. These findings are aligned with results from previous studies showing that women often experienced negative emotions from mastectomy-related physical changes and the feeling of incompleteness without a breast [7, 22–24]. Many participants mentioned that mastectomy was a distressing experience and they experienced mirror trauma; hence they avoided looking at themselves in the mirror or during showers [25–27]. Mirror trauma is the shock or severe discomfort in seeing one’s body in a mirror, often experienced when seeing or perceiving a dramatic change in one’s body [27]. These findings could be due to inadequate preparation for this difficult experience and insufficiency of psychological care support due to the unavailability of multidisciplinary oncological care approach in Syria. These findings are consistent with previous studies [8, 25–27], suggesting the need to provide these women with good preparation and enough psychological support to facilitate the acceptance and coping with their new body image and avoid the trauma of mirror viewing. It is crucial, therefore, to involve psychologists alongside oncologists in the treatment plan to help patients accept their illness and cope with the consequences of treatment [28]. The findings also showed that the participants experienced some change in their sexual life and relationships after conducting the mastectomy. This result is consistent with previous studies showed that mastectomy can adversely affect young women with breast cancer and their partners [13, 17, 18].

Various coping mechanisms were employed by the patients to handle stress, with social support and spiritual practices being the most common strategies used. Similar to other similar societies [8], Syrian society is known for its strong familial ties and family typically provides psychological, social, and financial support to their members facing challenges such as cancer. Furthermore, all participants in this study were Muslim and hold strong belief in God’s support to help them to accept their disease and treatment including finding solace and reassurance in reading the Quran and offering prayers [8, 13, 25].

Unfortunately, the results also showed that many women in this study experienced societal negativity towards breast cancer as this disease remains a stigma in many cultures, including the Arabic cultures [12, 13, 24]. Some participants expressed a strong desire for breast reconstruction to improve their appearances and self-confidence, particularly for the younger women who are harboring the prospect for future marriage. Indeed, breast reconstruction has been linked to improved quality of life for patients with breast cancer [29]. However, many Syrian women, affected by the ongoing crisis in the country since 2011, face financial barriers to reconstruction. With limited income, the high cost of the procedure is prohibiting young Syrian breast cancer patients grappling with this dual burden resulting from their diagnosis at a young age and the challenging living conditions. The ongoing war crisis has affected the accessibility of oncological medications, increased living costs, and has disturbed the equilibrium between income and expenses. As a result, patients prioritize treatment, family needs, and children’s education over breast reconstruction.

This study has several pertinent limitations that should be mentioned. First, purposive sampling was used in this study. While purposive sampling was intentional in qualitative research like this, it can also be a limitation because the sample might not be representative of the broader populations. Second, the interviews were conducted in Arabic and then translated into English by the first author for analysis. Translation can introduce potential biases or misinterpretations. Third, the interviews were conducted physically in a private room at the participant’s home. While physical sessions like these might make the participants feel comfortable, it might also introduce social desirability bias as participants might provide answers that they believed were socially acceptable in their home setting.

Despite its limitations, this study has identified important points, that if addressed, can enhance the care given to young females with breast cancer in conflict-ridden countries. For example, pre-habilitation programs that cater to both the psychological and physical needs of these women should be introduced. These programs would help prepare them to cope with the physical, psychological, and social challenges associated with mastectomy. Sharing images and videos of post-mastectomy outcomes can also help these women in coming to terms with their changed body. Furthermore, setting up sexual health counseling units for these women and their partners can help them fostering healthier sexual relationships and avoid a lot of sexual issues in future. The implementation of educational programs on TV and other platforms can increase awareness of breast cancer and reducing societal stigma experienced by these women.

## Conclusion

Mastectomy is a traumatic experience for many young women with breast cancer, affecting them psychologically, physically, and socially. The ongoing Syrian war crisis has further exacerbated these issues, as these women are unable to access breast reconstruction due to high costs of treatment, leaving them more vulnerable to societal stigma. Nurses, health professionals, and organizations should understand these negative ramifications of mastectomy as well as the need to devise strategies for raising breast cancer awareness, encouraging early detection, and promoting less aggressive treatments like conservative surgery, which may improve women’s quality of life.

## Data Availability

The transcripts may be obtained by contacting the corresponding author.
